# Pneumatosis Intestinalis Associated with Juvenile Dermatomyositis

**DOI:** 10.1155/2016/6497357

**Published:** 2016-05-08

**Authors:** Takako Miyamae, Naoko Ishiguro, Maria Yonezawa, Katsutoshi Tokushige, Hisashi Yamanaka

**Affiliations:** ^1^Institute of Rheumatology, Tokyo Women's Medical University, No. 10-22, Kawadacho, Shinjuku-ku, Tokyo 162-0054, Japan; ^2^Department of Pediatrics, Tokyo Women's Medical University, No. 8-1, Kawadacho, Shinjuku-ku, Tokyo 162-0054, Japan; ^3^Department of Dermatology, Tokyo Women's Medical University, No. 8-1, Kawadacho, Shinjuku-ku, Tokyo 162-0054, Japan; ^4^Institute of Gastroenterology, Tokyo Women's Medical University, No. 8-1, Kawadacho, Shinjuku-ku, Tokyo 162-0054, Japan

## Abstract

We herein report a case of pneumatosis intestinalis (PI), a condition characterized by the presence of gas within the wall of the digestive tract, associated with juvenile dermatomyositis (JDM). A 16-year-old girl, diagnosed with JDM at the age of 10, presented with abdominal pain and distention. She developed PI based on radiological findings that also included a dilated large intestine, extraluminal gas, and secondary diaphragmatic elevation. She was observed with medical therapy including bowel rest and hyperbaric oxygen therapy. However, she ultimately developed a strangulated obstruction 5 years after presentation with PI and large intestine resection and colostomy were performed emergently.

## 1. Introduction

Juvenile dermatomyositis (JDM) is an autoimmune vasculopathy of uncertain origin involving multiple organ systems. It is defined as chronic inflammation of the striated muscle and skin [1, 2]. Classic JDM presents with an insidious progression of malaise, fatigue, muscle weakness, fever, and a rash that may predate diagnosis by three to six months. In more than three-fourths of the children with JDM [3], the cutaneous abnormalities are pathognomonic of the disease at presentation. The most typical cutaneous manifestations are heliotrope discoloration of the upper eyelids and Gottron papules more frequently in young children. In cases with severe skin vasculopathy, cutaneous ulcers present in 5% to 10% of patients [4, 5].

Gastrointestinal manifestations may also be present as extra-muscular symptoms in JDM. Vasculopathy in the mucosa of the gastrointestinal tract with resulting tissue ischemia or an acute mesenteric infarction can occur [6].

Pneumatosis intestinalis (PI), first described in 1754, is a rare condition characterized by gas bubbles within the wall of the digestive tract. It has been associated with medical conditions including gastrointestinal and chronic pulmonary diseases [7]. However, it rarely occurs with autoimmune disorders. Among several PI cases associated with JDM, their gastrointestinal severity varies from benign to life-threatening causes [8]. We experienced a case of JDM complicated by PI eventually requiring a colostomy.

## 2. Case Presentation

In 1998, a girl presented with facial erythema and Gottron's papules at 10 years of age and visited the Department of Dermatology at Tokyo Women's Medical University. Muscle weakness and elevation of muscle-derived enzymes (creatine kinase 1105 IU/L, aldolase 30.9 IU/L, and myoglobin 470 ng/mL) developed during the following year. She was diagnosed as having JDM on clinical and pathological grounds after skin and muscle biopsy. Muscle biopsy revealed perivascular mononuclear inflammatory infiltrate, arterial wall thickening, and perifascicular atrophy. Anti-Jo-1 antibody was negative. Oral prednisolone (PSL, 60 mg/day, 2 mg/kg) alleviated the clinical symptoms and laboratory findings. Following a decrease in the dosage of PSL, myositis and skin erythema developed at age 14. The myositis remained stable whereas the skin rash intermittently progressed.

**Figure 1:**
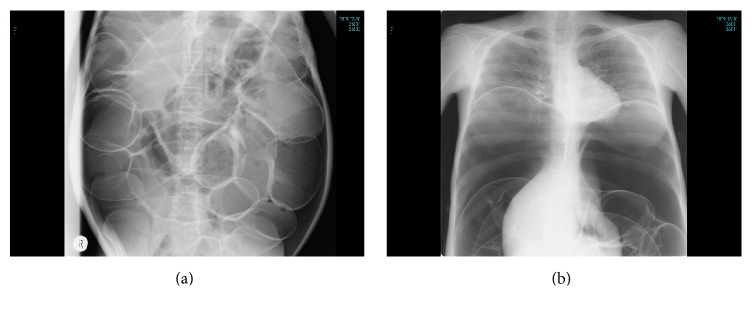
Plain radiographs ((a) lateral view of the abdomen and (b) frontal view of the chest and abdomen) show extensive gas in the colon wall, a dominance of air in the abdominal cavity above the colon, and secondary diaphragmatic elevation.

She presented with abdominal pain and severe distention when the dosage of PSL was decreased to 9 mg/day in 2004, at the age of 16. She was afebrile. Radiological examination demonstrated pneumatosis, a dilated large intestine, and dominance of air in the abdominal cavity suggesting the bowel-wall air above the colon (Figures 1(a) and 1(b)). She was not able to lie down on her back and developed respiratory distress because of the marked abdominal distention and secondary diaphragmatic elevation. She was diagnosed with PI and treated medically with bowel rest and hyperbaric oxygen therapy. She partially improved but abdominal pain and distention persisted, sometimes got a little better but then got worse again, and finally developed large bowel obstruction with strangulation in 2009 necessitating emergent large and small bowel resection with a double barrel colostomy. Further exploration revealed foci of necrosis with several perforations in both large and small intestines. Since the colostomy was performed, she was then receiving nutrition parenterally and has been free from abdominal pain with less distention without hyperbaric oxygen therapy during 6 years after the resection. Her steroids were changed from oral PSL to intravenous betamethasone at a dosage of 0.3 to 0.9 mg/day depending on skin manifestations but not on abdominal symptoms. In February 2015, anti-aminoacyl-tRNA synthetase antibodies were not detected and the patient was diagnosed with severe osteoporosis (% of young adult mean bone marrow density between lumbar vertebrae L2–L4: 53%), presumably caused by long term administration of betamethasone. She was then partially switched back to oral PSL. The dosage of PSL has been controlled based on gastrointestinal and skin manifestations and is currently maintained at 10 mg daily.

## 3. Discussion

We reported a case of PI associated with JDM eventually requiring large bowel resection and colostomy. PI is not a diagnosis but rather a physical or radiographic finding, usually as a result of a pathologic process. Impaired mucosal integrity, increased intraluminal pressure, bacterial flora, and intraluminal gas during illness all can lead to the presence of gas within the wall of the gastrointestinal tract [9].

Among autoimmune disorders, PI is commonly noted in systemic sclerosis but rarely associated with dermatomyositis or JDM. Previously, 8 cases of PI in JDM have been reported in the literature (Table 1). Two patients died of intestinal perforation and subsequent sepsis [10, 11]. The time required to normalization of gastrointestinal conditions varied from a week to over a year in those patients that survived. This patient had 5 years from the time of first manifestations of PI until the development of a strangulated obstruction and was never fully healthy during that time. Intestinal resection and colostomy were thought to be unavoidable.

In the JDM cases that developed PI, skin manifestations were more common than myositis. Two of the 8 cases had skin ulceration. The pathogenesis of PI in JDM is not clear. However, some authors have speculated that gastrointestinal ischemia secondary to vasculopathy could result in loss of integrity of the mucosa and bacterial production of gas with submucosal dissection of intraluminal gas [12]. The high frequency of skin manifestations and ulcers might suggest that skin vasculitis has a correlation with vasculopathy in the mucosa of the gastrointestinal tract. Interestingly, severity and prognosis of PI associated with JDM vary though the number of reported patients was small. Benign cases were diagnosed by routine X-ray finding without abdominal manifestations and resolved in natural course; however, there were fatal cases complicated with intestinal perforation, subsequent infection, and sepsis [Table 1]. The difference of the prognosis of PI is unclear but can be affected by severity of vessel vasculopathy, initial treatment, and myositis specific/associated antibodies.

Immunosuppressive treatments including glucocorticoids seemed to be effective in some of the reported cases and these were able to suppress the vasculopathy in the early phase of PI. In addition, they could help to cause the submucosal lymphoid tissue atrophy and subsequent dissection of air into the bowel wall that occurs. However, once peritonitis and sepsis occur, the problem is surgical. Considering the fact that most of the cases were reported before 2000, early diagnosis and timely initiation of immunosuppressive therapy may help to prevent vasculopathy and the development of PI. PI should be considered on the differential diagnosis in patients with JDM presenting with abdominal complaints, especially in patients with severe skin vasculitis.

## Figures and Tables

**Table 1:** Summary of reported cases of pneumatosis intestinalis associated with juvenile dermatomyositis.

	Onset age of PI	Period from JDM onset to PI onset	Treatment before PI onset	Manifestations of PI	Image findings and clinical diagnosis	Disease activity of JDM at PI onset	Clinical course of PI	Outcome
1 [13]	4.8, female	1 Y	GCs	Abdominal pain, diarrhea, constipation, and movable mass in the left lower quadrant	Cystoid gas collection in the mid transverse colon and splenic flexure	Calcinosis universalis was noted	Pneumatosis persisted for 25 months	Survived

2 [14]	8.5, female	3 Y	GCs, AZP, CY, and MTX	Abdominal pain and abdominal distention	Gas-filled hepatic flexure of colon and extraluminal gas	Refractory myositis and skin manifestations with disseminated subcutaneous calcification	Abdominal manifestations remitted during the next 10 days with PSL and MTX. Intramural gas persisted for four months	Survived

3 [15]	12, female	3 Y	None	Dysphagia	Cystoid gas collection	Noted myositis and dermatitis	Treated with oral GCs	Survived

4 [10]	8.5, female	1 Mo	GCs and MTX	Abdominal pain, vomiting, diarrhea, and fever	Perforation and peritonitis	Increased muscle weakness, rash, and rising muscle enzyme values recurred	Died 6 weeks after the development of PI owing to complications of perforations, peritonitis, and candida sepsis	Died

5 [11]	11, female	3 Mo	GCs and CY	Abdominal pain, bilious emesis, and fever	Extensive extraluminal gas collection in the right abdomen and flank. Peritonitis, retroperitoneal abscess, and duodenal perforation were found at laparotomy	Became bedridden because of progressive muscle weakness from JDM onset	Discharged 8.5 months after admission over multiple episodes of sepsis and 8 laparotomies	Survived

6 [11]	15, male	2 Y	GCs	Abdominal discomfort, pain, vomiting, fever, and hematemesis	Following appendicitis and appendectomy, PI occurred with duodenal perforation and peritonitis	Exacerbated muscle weakness two months previously	Died of perforation, sepsis and multiple organ failure on the 21st hospital day despite four laparotomies	Died

7 [12]	7, female	3 Mo	GCs and MTX	Abdominal pain	Intramural air in the ascending and transverse colon	Prominent skin rash and vascular ulcers in the axillar, minimal proximal muscle weakness	Treated with intravenous antibiotics and parental nutrition. Clinical improvement evident after a week	Survived

8 [8]	8, female	1 Mo	GCs, MTX, and mPSL pulse	Cough and abdominal distention	CT showed extensive PI in the large colon	Increased weakness and a vasculitic ulcer on upper eyelid and in the nare	Treated with intravenous antibiotics and a short period of bowel rest	Survived

Present case	16, female	6 Y	GCs	Abdominal pain and abdominal distention	Extensive gas-filled colon and extraluminal gas above colon	Intermittently worsened skin manifestations	Complicated with strangulated obstruction and large intestine resection and colostomy were performed	Survived

PI: pneumatosis intestinalis, JDM: juvenile dermatomyositis, Mo: month(s), Y: year(s), GCs: glucocorticoids, PSL: prednisolone, CY: cyclophosphamide, AZP: azathioprine, MTX: methotrexate, and mPSL: methylprednisolone.
